# An effective treatment and suspicious adverse reaction to Ibrutinib in a patient diagnosed with splenic B-cell lymphoma/leukaemia with prominent nucleoli: A first case report

**DOI:** 10.1097/MD.0000000000036022

**Published:** 2023-12-29

**Authors:** Mei-Xiao Shen, Fu-Ling Li, Xian-Sheng Luo, Zhi-Ming Wang

**Affiliations:** a Department of Hematology, Affiliated Haikou Hospital of Xiangya Medical College, Central South University, Haikou, China; b Department of Pharmacy, Affiliated Haikou Hospital of Xiangya Medical College, Central South University, Haikou, China.

**Keywords:** aortic dissection, B-cell prolymphocytic leukemia, BTK inhibitor, ibrutinib, splenic B-cell lymphoma/leukaemia with prominent nucleoli

## Abstract

**Rationale:**

Splenic B-cell lymphoma/leukemia with prominent nucleoli (SBLPN) is a new classification, which is so rare that it lacks clinical data.

**Patient concerns:**

An increased proportion of prolymphocytes (84%) in the bone marrow smear. Whole exon sequence analysis revealed a TP53 mutation.

**Diagnoses:**

Combining the clinical features with laboratory test results led to a diagnosis of SBLPN which was made according to the 5th edition of the WHO classification of hematolymphoid tumors, although the patient was diagnosed with B-PLL when guided by the 4th edition of the WHO classification.

**Interventions:**

The use of Ibrutinib as an effective treatment.

**Outcomes:**

The patient was in complete remission after 5 months of Ibrutinib and then died of sudden aortic dissection.

**Lessons:**

Ibrutinib was an effective regimen for SBLPN. Aortic dissection might be considered as a suspicious adverse reaction to Ibrutinib.

## 1. Introduction

B-cell prolymphocytic leukemia (B-PLL) is a rare B-cell chronic lymphoproliferative disorder (BCLPD), accounting for < 1% of lymphoid leukemias.^[1]^ The median age at the time of B-PLL diagnosis is 69 years while the median overall survival period is approximately 3 years.^[1]^ About 10% to 15% of patients are asymptomatic at the time of diagnosis, and the disease can remain latent for several years. However, most patients present aggressive bio-clinical features with B symptoms (fever, night sweats, and unintentional weight loss), massive splenomegaly, absent lymphadenopathy, and obvious lymphocytosis (lymphocyte count > 100 × 10^9^/L).^[2]^

B-PLL was previously recognized as a distinct mature B-cell entity in the 2016 version of the WHO Classification of Haematolymphoid Tumours^[3]^ but has since been removed from the 2022 edition.^[4]^ B-PLL is classified into 3 closely related diseases: Lymphomas that harbor the IGH/CCND1 fusion rearrangement are classified as variant mantle cell lymphoma (MCL); CD5-positive non-mantle B-cell lymphoma with ≥ 15% prolymphocytes content is now classified as prolymphocytic progression of chronic lymphocytic leukaemia (CLL), and; Other cases are classified into splenic B-cell lymphoma/leukaemia with prominent nucleoli (SBLPN).^[3]^ Here, we report a first case of an individual initially diagnosed as B-PLL but whose diagnosis was revised to SBLPN based on the new classification. The newly classified SBLPN is so rare that it lacks clinical data. Thus, we examined the heterogeneity, new classification, and treatment regimens for B-PLL.

## 2. Case description

**Figure 1.**
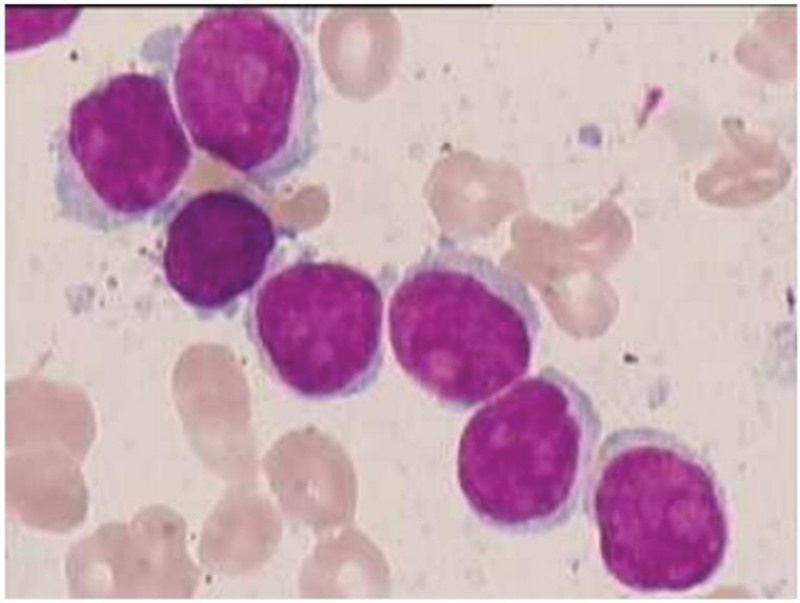
Bone marrow aspirate showing 84% medium size prolymphocytes with basophilic cytoplasm, partially condensed chromatin and a prominent nucleus (May–Grunwald–Giemsa stain, 100 × magnification).

A previously healthy 63-year-old male, presenting with fever, cough, and shortness of breath, was admitted to the First Hospital of Jilin University, Changchun, Jilin Province, China in July 2018. A complete blood cell analysis revealed leukocyte counts of 102.37 × 10^9^/L, lymphocyte counts of 90.08 × 10^9^/L (prolymphocytes accounting for 80% of the total counts), hemoglobin level of 68 g/L, and platelet counts at 115 × 10^9^/L. No obvious abnormality was observed from the patient’s electrocardiogram, echocardiograph, abdominal ultrasonography examination, and renal liver function tests. Monoclonal immunoglobulins were not detected in the peripheral blood. The bone marrow smear revealed that a diffuse bone marrow infiltration, with an increased proportion of lymphocytes (87%), of which 84% were prolymphocytes. The prolymphocytes, presented as round or oval cells (at twice the size of a typical lymphocyte), with a basophilic cytoplasm while the nucleus showed partially condensed chromatin and was round or oval shaped with a prominent nucleus (Fig. 1). Granulocytic, erythrogenic, and megakaryocytic lineages were suppressed. Flow cytometry analysis revealed that abnormal lymphocytes occupied 80% of nucleated cells, and mainly expressed CD19, CD20, CD22, CD79b, FMC7, CD5, mKappa (strong) but did not express CD10, CD200, CD11c, CD43, CD23, CD7, CD34, CD123, CD103, CD25, mLambda, cKi67, ZP70. Whole exon sequence analysis revealed a TP53 mutation, NM_00546: c. 920-1G > A within exon 9 at a frequency of 51.9% (based on a sequencing depth of 2372X). Polymerase chain reaction analysis of 17 lymphocytic leukemia related fusion genes was negative but chromosomal analysis established that the genotype was 43 to 45, X, Y, del(7q), add(8p), add(12p), −13, −14, add(15p), −17, −18, +marx4[cp6]/46, XY ^[6]^. Immunoglobulin variable heavy chain rearrangement was positive, and T-Cell Receptor rearrangement was detected by polymerase chain reaction while IgH/CCND1 was not detected by Fluorescence In Situ Hybridization. A bone marrow biopsy subjected to hematoxylin and eosin staining and Periodic-Acid Schiff staining revealed that bone marrow proliferation was extremely active (90%). In addition, there was irregular distribution of heterogeneous lymphocytes with varying cell sizes, limited cytoplasm and round or irregular nucleus. The matured granulocytic and erythrogenic lineage cells were scattered and were abundant with megakaryocytes while lobulated nuclei were dominant and reticular fiber staining was at grade 1. Further immunohistochemistry was able to detect the presence of CD20+, PAX5+, CD5−, CD10−, CylinD1−, SOX-11−, Ki-67 + individual cells, CD23−, CD25−, CD11c−, and CD3−. Physical examination of the patient revealed hepatomegaly (not palpable) and splenomegaly (I line 10 cm, II line 16 cm, III line + 2 cm), but no general enlargement of the lymph nodes. The abdominal ultrasonography showed that the maximum oblique diameter of right lobe of the liver was 149mm, while the spleen was 222*77mm in size. Combining the clinical features with laboratory test results led to a diagnosis of SBLPN which was made according to the 5^th^ edition (2022) of the WHO Classification of Haematolymphoid Tumours,^[4]^ although the patient was diagnosed with B-PLL when guided by the 4th edition (2016) of the WHO Classification.^[3]^ The main complications were pulmonary infection and acute left heart failure. Following the diagnosis, the patient refused chemotherapy and instead opted for intermittent blood transfusions and herbal treatment, both of which proved ineffective. Then the patient was administrated 1 course of R-CVP (Rituximab, Cyclophosphamide, Vincristine, Prednisolone) and 2 courses of R-CHOP (Rituximab, Cyclophosphamide, Doxorubicin, Vincristine, Prednisolone) chemotherapy regimen separately between November 2019 and January 2020. Following the treatment, the maximum oblique diameter of right lobe of the liver was 142 mm, the spleen was in the size of 195*72 mm, the blood cell counts improved with leukocytes at 22.66 × 10^9^/L, lymphocyte counts of 4.01 × 10^9^/L, hemoglobin level was at 132g/L and platelets at 104 × 10^9^/L, which indicated a partial response with 10% of B prolymphocytes in the bone marrow. However, after 3 cycles of chemotherapy, the patient chose to discontinue treatment against medical advice.

**Figure 2.**
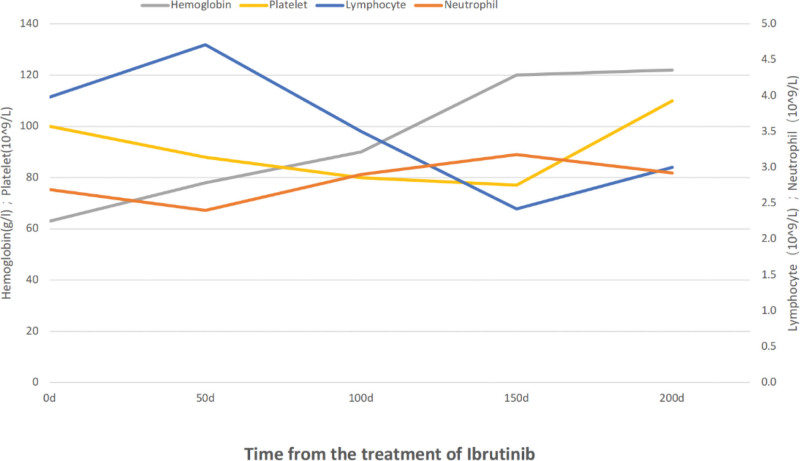
Complete blood cell profiles over the course of Ibrutinib treatment.

**Figure 3.**
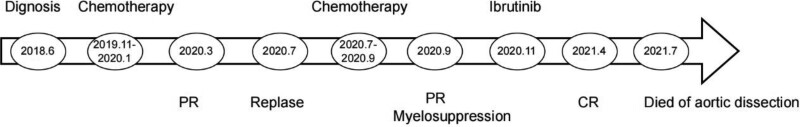
Schematic of the clinical course of treatment given to the patient. CR = complete response, PR = partial response.

In July of 2020, the patient again presented with weakness and dizziness and was admitted to the hospital. Complete blood cell counts were recorded as leukocytes: 90.96 × 10^9^/L, 59% prolymphocytes, reticulocytes at 59 × 10^9^/L, hemoglobin level of 48 g/L and platelet count at 134 × 10^9^/L. In addition, a Coomb test was positive, haptoglobin was 6 mg/dL, lactic dehydrogenase level was 288 u/L, total bilirubin was 54 umol/L, and indirect bilirubin was 34 umol/L. Bone marrow cell morphology showed a diffuse bone marrow infiltration with prolymphocytes accounting for 57.5% of total cells suggesting B-cell prolymphocytic leukemia relapse accompanied by autoimmune hemolytic anemia. The patient was administrated 3 courses of R-CHOP chemotherapy from July 2020 to September 2020, following which, he developed grade D myelosuppression (neutrophil counts of 0.47 × 10^9^/L, and platelets at 80 × 10^9^/L), presented with pulmonary infection and heart failure. After receiving empirical anti-infection and cytokine treatment, the patient recovered and refused further chemotherapy (including Rituximab). In November 2020, bone marrow aspirates showed 10% prolymphocytes, indicating a partial response, while Eastern Cooperative Oncology Group performance status was 3. He was administrated Ibrutinib (420 mg/day) orally without any adverse side effects. After 5 months, complete blood cell counts, bone marrow aspirates and flow cytometry showed complete remission. The abdominal ultrasonography showed that maximum oblique diameter of right lobe of the liver was 130 mm, while the spleen was 118 × 65 mm. The patient remained stable without recurrence and then died of sudden aortic dissection finally in July 2021. The complete blood cells data is shown in Figure 2. The clinical treatment process for patients is shown in Figure 3.

## 3. Discussion

A combination of morphological observations and immunophenotyping techniques are required to confirm a diagnosis of B-PLL from other BCLPD. The morphology of prolymphocytes is key to an accurate diagnosis whereby the cells are twice the size of a normal lymphocytes with a prominent central round nucleus, moderately condensed nuclear chromatin, and a relatively small amount of indistinct basophilic cytoplasm. B-PLL immunological markers largely overlap with other BCLPD and include expressed surface membrane immunoglobulin (strong), CD19+, CD20+, CD22+, CD79a + and FMC7 + (CLL score 0–1), and the absence of or weekly expressed CD23 and CD5. Cytogenetic analysis can reveal abnormal complex karyotypes including losses of 6q, 11q, 13q and 17p-.^[1]^ Molecular assays were used to demonstrate that TP53 mutations and C-MYC overexpression were significantly present in more than half of B-PLL patients.^[5]^ The presence of TP53 mutations is coupled with resistance to chemotherapy and short survival times and is associated with poor prognosis.

To assess a positive diagnosis is challenging because of an overlap in clinical presentation and laboratory test results with other BCLPD. Differential diagnosis is usually based on several factors: B-PLL cells are large B-lymphocytes with a prominent central round nucleolus. Moreover, CLL/small lymphoma leukaemia (SLL) cells are small lymphocytes with a nucleus, although CLL/SLL is identified by the presence of 10% to 55% prolymphocytes in peripheral blood.^[6]^ In the case described here, the proportion of prolymphocytes was higher than 55%, with a CLL score of 1, which did not support a diagnosis of CLL/SLL. However, cases that were classified as CLL/SLL based on the presence of ≥ 15% prolymphocytes as outlined in the 2016 version of the WHO Classification, are now classified as prolymphocytic progression of CLL in line with the 2022 version of the WHO Classification;^[3,4]^ The absence of cylinD, IgH/CCND and sox-11 can assist in differentiating B-PLL from MCL. However, some reports have identified B-PLL as a subtype of MCL^[7]^ and this led to B-PLL harboring at (11; 14) abnormality being classified as MCL in the 2022 (5^th^ edition) WHO Classification;^[4]^ The absence of cytoplasmic hairy projections and villi would exclude hairy cell leukemia variants, while the negative detection of both CD25 and CD103 would exclude splenic marginal zone lymphoma.

The “wait and watch” approach is recommended for indolent asymptomatic patients.^[2]^ Howerver, standard therapeutic regimens would not be available for a rare disease that lacks data from prospective comparative studies. As the typical clinical features of B-PLL are similar with other BCLPD, standard BCLPD-targeted therapeutic regimens that are applied to B-PLL patients are only partly effective and patients relapse within a short period. Previously a purine analogue-based regimen was recommended to 8 patients and 5 of the 8 patients achieved complete remission, with a median response duration of 14 months.^[8]^ The CVP containing therapies was effective in roughly 50% of a group of 23 B-PLL patients.^[9]^ Allo-hematopoietic stem cell transplantation (HSCT) remains an uncertain strategy to achieve a long-term response and is also not feasible for all patients due to age-related efficacy issues. Kalaycio et al^[10]^ reported a median progression-free survival of only 3.5 months for 11 B-PLL patients who received allo-HSCT. Allo-HSCT is administered on its own as a salvage therapy for young patients, those harboring TP53 disruptions, patients who respond poorly to B-cell receptor (BCR) inhibitors or patients who previously relapsed after responding to other forms of salvage chemotherapy.^[2,10,11]^

The presence of TP53 mutations is reported to be the main prognostic factor associated with resistance to conventional therapies and short survival times.^[12,13]^ Ibrutinib covalently bound to cysteine 481 in the ATP binding pocket of Bruton tyrosine kinase, resulting in the inhibition of BCR activation and the induction of apoptosis in mature B-cell maglignancies.^[14]^ Data showing the efficacy of BCR inhibitors in the presence of TP53 mutations is inferred from CLL patients.^[15,16]^ Gordon et. al. demonstrated that in 2 cases of B-PLL with TP53 dysfunctions, when the patients were treated with Ibrutinib as second-line therapy, the tumor burden decreased.^[17]^ Moore et al^[18]^ reported that in 6 patients receiving a treatment regimen containing Ibrutinib as initial therapy for B-PLL, the response rate was 83.3%, the median progression-free survival was 34.7 months (range 2.6–50.5 months), but the median overall survival was not available. Based on case reports of successful Ibrutinib treatment of B-PLL as the upfront or second-line therapy,^[17–20]^ Ibrutinib could play an important role in the management of this rare disease.

Three lines of evidence support the association between ibrutinib usage and the occurrence of dissecting aortic aneurysm events. First, there was a clear temporal relationship between the initiation of Ibrutinib treatment and the onset of aortic dissection. Second, the patient had no hypertension in his past medical history nor during administration. Aortic dissection was not visible on the chest CT scan prior to initiating treatment with Ibrutinib. Thus, there were no confounding causes or alternative explanations that could clarify the onset of aortic dissection. Up to September 30, 2022, only 10 cases of aortic dissection were listed as a suspicious adverse reaction to Ibrutinib in the FDA Adverse Event Reporting System. Eight of 10 patients were also afflicted by other underlying diseases (including carcinomas, cardiovascular diseases, cerebrovascular diseases and had recently undergone surgery), suggesting possible drug interactions contributed to adverse reaction to Ibrutinib. Only 2 patients were free of other co-morbidities but still presented with aortic dissection. A search within the pharmacovigilance database Vigibase identified a further 12 cases of suspect Ibrutinib related aortic dissection that occurred unexpectedly but none of those individuals had any record of primary diseases or relating information available in the database. Nonetheless, this report presents the first published case of aortic dissection in a patient treated with ibrutinib.

The most common Ibrutinib-associated markers of cardiac toxicity are hypertension and cardiac arrhythmias. Cardiotoxicity of anticancer therapies may result from the “on-target” effects of vascular endothelial growth factor (VEGF) inhibition or “off-target” effects resulting from inhibition of tyrosine kinases.^[21]^ Hypertension appears to be a predisposing factor for aortic dissection, however, it is not an essential factor. For the patient in this study, the evidence might support the suggestion that Ibrutinib could be a contributing factor towards aortic dissection. We also suggest that a possible mechanism is the off-target anti-VEGF activity of Ibrutinib. Blocking of VEGF pathway may contribute to arterial wall injury resulting in conditions that favor artery dissections or aneurysms by; the inhibition of nitric oxide synthesis; The rarefaction of tissue capillaries and; Vascular aging.^[22]^ To date, the mechanism involved that links aortic dissection with Ibrutinib is unclear and needs to be further investigated. Aortic dissection is a sudden life-threatening event with high fatality rates. This case report is intended to increase awareness among physicians of the possible complications of Ibrutinib treatment and the need to monitor patients appropriately during Ibrutinib administration.

## 4. Conclusion

In conclusion, SBLPN is a rare B-cell lymphoma and lacks effective treatments. Here, we describe the first case of a SBLPN patient successfully treated with Ibrutinib but who unfortunately died of aortic artery dissection. In the future, investigations into the effectiveness of BCR inhibitors as a single agent regimen for SBLPN patients should be extended, especially for the group of patients harboring TP53 mutations. Furthermore, the possible “off-target” anti-VEGF activity of Ibrutinib may contribute to aortic dissection, which seems to be a serious adverse reaction to ibrutinib.

## Acknowledgements

The authors would like to express their gratitude to EditSprings (https://www.editsprings.cn) for the expert linguistic services provided.

## Author contributions

**Visualization:** Fu-Ling Li, Zhi-Ming Wang.

**Writing – original draft:** Meixiao Shen, Xian-Sheng Luo.
